# A Smartphone App (PRIMI) to Promote Healthy Diet, Physical Activity, and Health Literacy After Childbirth Among Migrant Women: Protocol for a Randomized Controlled Trial

**DOI:** 10.2196/79277

**Published:** 2025-10-17

**Authors:** Anna Seiterö, Maryam Shirvanifar, Marie Jubran Leksell, Maria Rydfjord, Baydaa Al-saedi, Aisha Salah Ahmed, Tayebeh Gharakhani Bahar, Daniel Berglind, Hanna Henriksson, Viktor H Ahlqvist, Josefin Wångdahl, Ulrika Müssener, Pontus Henriksson

**Affiliations:** 1 Department of Health, Medicine and Caring Sciences Linköping University Linköping Sweden; 2 Refugee Health Centre Region Östergötland Norrköping Sweden; 3 Department of Obstetrics and Gynecology Region Kalmar County Kalmar Sweden; 4 Department of Global Public Health Karolinska Institutet Stockholm Sweden; 5 Centre for Epidemiology and Community Medicine Region Stockholm Stockholm Sweden; 6 Center for Wellbeing, Welfare and Happiness Stockholm School of Economics Stockholm Sweden; 7 Unit for Strategic Healthcare Region Östergötland Linköping Sweden; 8 Department of Biomedicine Aarhus University Aarhus Denmark; 9 Institute of Environmental Medicine Karolinska Institutet Stockholm Sweden; 10 Aging Research Centre Karolinska Institutet and Stockholm University Stockholm Sweden; 11 Division of Nursing Department of Neurobiology, Care Sciences, and Society Karolinska Institutet Stockholm Sweden; 12 Department of Public Health and Caring Sciences Uppsala University Uppsala Sweden

**Keywords:** mobile health, mHealth, telemedicine, behavior change, emigrants and immigrants, postpartum, diet, physical activity, randomized controlled trial

## Abstract

**Background:**

Migrant health, including reproductive health, is an important public health priority. The postpartum period is a critical window for establishing healthy behaviors that can impact long-term health. Mobile health interventions offer a scalable solution, but existing tools are often not culturally or linguistically adapted for diverse populations. To the best of our knowledge, no previous study has evaluated the effectiveness of a culturally targeted mobile health intervention delivered after childbirth to promote a healthy diet and physical activity among migrant women.

**Objective:**

The PRIMI (Promoting Reproductive Health in Migrant Women) trial will determine the effectiveness of a smartphone app (the PRIMI app) on primary (diet quality and moderate-to-vigorous physical activity) and secondary (health literacy, BMI, self-efficacy, and self-rated health) outcomes in first-generation migrant women after childbirth.

**Methods:**

A 2-arm randomized controlled trial will be conducted to examine the effectiveness of the PRIMI app. First-generation migrant women who have given birth within 6 months, are aged 18 years or older, and prefer to receive health-related information in any of the provided languages are eligible to participate in the study and will be recruited through health care services in Sweden. The women will be randomized to the control group (standard care, eg, parental guidance and support within child health care) or the intervention group (PRIMI app+standard care) in a 1:1 ratio. A total of 200 women (100 per group) will be included in the study. A waitlist control strategy will be applied so that women in the control group will receive the PRIMI app after the follow-up measurement at 6 months. Outcomes will be assessed at baseline and at the 6-month follow-up. The PRIMI app, developed within the PRIMI project, is compatible with both Android and iOS. It contains weekly changing themes focusing on physical activity, diet, and health literacy throughout the 6-month intervention period. The app integrates behavior change techniques such as feedback and monitoring, goal setting, and instructions on how to perform the behavior. The app’s language can be switched among 4 common languages (Arabic, Somali, English, and Swedish), and all textual content can be accessed through audio files for listening. All procedures have been approved by the Swedish Ethical Review Authority (reference 2022-06733-01 and 2024-00135-02).

**Results:**

Recruitment of study participants is planned to begin in September 2025. We anticipate completing recruitment in 2026 and that the results of the PRIMI trial will be available in 2027.

**Conclusions:**

This study will provide novel evidence on the effectiveness of the PRIMI app in promoting healthy behaviors and supporting postpartum health among migrant women. This is highly relevant given the lack of previous comparable studies and the urgent need for tailored postpartum interventions for migrant populations.

**Trial Registration:**

ClinicalTrials.gov NCT06881277; https://clinicaltrials.gov/study/NCT06881277

**International Registered Report Identifier (IRRID):**

PRR1-10.2196/79277

## Introduction

### Background

International migrants are a growing population in many European countries, including Sweden. Currently, more than 25% of women giving birth in Sweden and more than 20% of women giving birth in Europe are first-generation migrant women [1]. Research suggests that migrant women may face higher risks of adverse health outcomes during and after pregnancy, although the extent of these inequalities may differ according to specific maternal birth regions and health outcomes [2-5]. Therefore, improving migrant health, including reproductive health, is an important public health priority [2,6].

The period after childbirth has been identified as a critical window to promote healthy diet, physical activity, and body weight as excessive postpartum weight retention may have long-lasting effects on later obesity, cardiovascular health, and the outcomes of subsequent pregnancies [7-11]. However, there is a lack of lifestyle interventions after childbirth that target migrant women [8,12,13]. Mobile health (mHealth) interventions have gained a lot of interest as a practical way to deliver lifestyle support, guidance, and individually tailored targets to improve diet quality, physical activity, and BMI [14,15]. However, while such mHealth interventions may provide accessible support regardless of migrant and socioeconomic status [16], concerns have been raised that mobile phone apps are not sufficiently tailored for culturally and linguistically diverse women [17]. This is despite the fact that adequate adaptation of language system and cultural values may increase user engagement and intervention effectiveness through mechanisms such as self-efficacy and health literacy [18]. Specifically, we are unaware of any lifestyle app provided by health professionals to women in Sweden after childbirth or of any scientifically evaluated app tailored to migrant women after childbirth worldwide that targets diet and physical activity. Thus, there is a great need for randomized controlled trials that examine the effectiveness of mHealth interventions to promote healthy diet and physical activity after childbirth in migrant women. This project, the PRIMI (Promoting Reproductive Health in Migrant Women) project, builds on our previous mHealth interventions targeting other populations (eg, pregnant women and adolescents) [19-21] to provide evidence on the effectiveness of a smartphone app on health behaviors after childbirth in migrant women.

### Objectives

The aim of this study protocol is to describe the study design and methods of the PRIMI trial. The trial has been registered on ClinicalTrials.gov (NCT06881277). The PRIMI trial will evaluate the effectiveness of a smartphone app (the PRIMI app) in addition to standard care as compared to standard care alone. The outcomes are (1) diet quality (primary outcome), (2) moderate-to-vigorous physical activity (primary outcome), (3) health literacy (secondary outcome), (4) BMI (secondary outcome), (5) self-efficacy (secondary outcome), and (6) self-rated health (secondary outcome) in first-generation migrant women after childbirth. We hypothesize that migrant women who are provided access to the PRIMI app will report better diet quality and more moderate-to-vigorous physical activity compared to those without access to the app.

## Methods

### The PRIMI App

#### Context

In Sweden, approximately 25% of women who give birth were born in another country [1]. Apart from Sweden, common maternal birth regions include North Africa and the Middle East (eg, Iraq, Syria, and Iran), sub-Saharan Africa (eg, Somalia, Eritrea, and Ethiopia), and Europe (eg, Poland and Ukraine) [5,22,23]. Although no official statistics are collected on the languages spoken in Sweden, Arabic, Somali, and English are among the most common languages spoken by first-generation migrant women in Sweden. In addition, nearly 90% of all foreign-born women in Sweden have access to a mobile phone [24].

#### Development of the App

**Figure 1:**
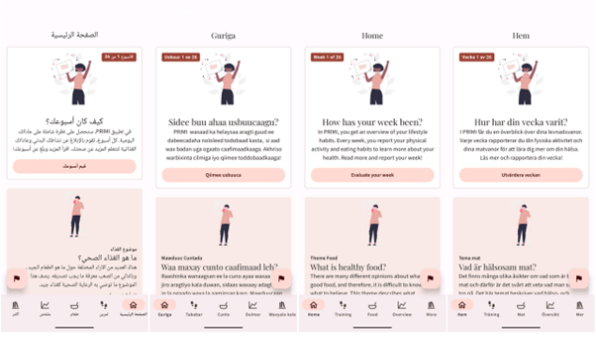
Screenshots of the home screen illustrating a notification for the weekly feedback and monitoring feature, as well as the weekly theme (focusing on diet, physical activity, or health literacy). All participants have access to all content in 4 languages: Arabic, Somali, English, and Swedish.

The PRIMI app is a fully automatic, culturally targeted, and comprehensive 6-month smartphone intervention aimed at improving diet quality, physical activity, and health literacy in migrant women. Screenshots of the PRIMI app are shown in Figure 1. Users can switch the app’s language among 4 options: Arabic, Somali, English, and Swedish. We included Swedish for women who prefer or have acquired sufficient Swedish language skills to use the app. All textual content can be read aloud through audio files. The PRIMI app is compatible with both Android and iOS and is available on the Google Play Store and Apple App Store for individuals with a study-specific user code.

A technical platform built specifically for the PRIMI app was used for its development, which took place in collaboration between the research team and the staff at the Digitalisation Division at Linköping University from 2024 to 2025. All decisions about the specific content and features of the PRIMI app were made by a multidisciplinary and multicultural team with expertise in nutrition, physical activity, behavioral medicine, and health literacy.

While our previous work [25] served as a model for the app’s overall structure, the target group was consulted before designing the app’s visual and textual content. Thus, individual interviews conducted with health professionals from maternity care and child health care centers, as well as migrant Arabic- and Somali-speaking mothers, informed decisions about the app’s content. For example, content was incorporated to address psychological well-being, including information on support groups within child health centers, as the qualitative data were related to loneliness and psychological distress after childbirth. In addition, the content was designed to align with the target group’s preference for relatable images and evidence.

The technical platform enabled the research team to continuously edit all content, including translations of texts and audio files into all targeted languages (Arabic, Somali, English, and Swedish). The development was an iterative process involving pilot-testing by collaborators with experience from the Refugee and Migrant Medicine Center in the Region of Östergötland, maternity care, and other health care settings. This was to improve usability and ensure that the app was relevant for the target group rather than for scientific purposes. Quality assurance was further conducted by Arabic- and Somali-speaking research team members.

**Figure 2:**
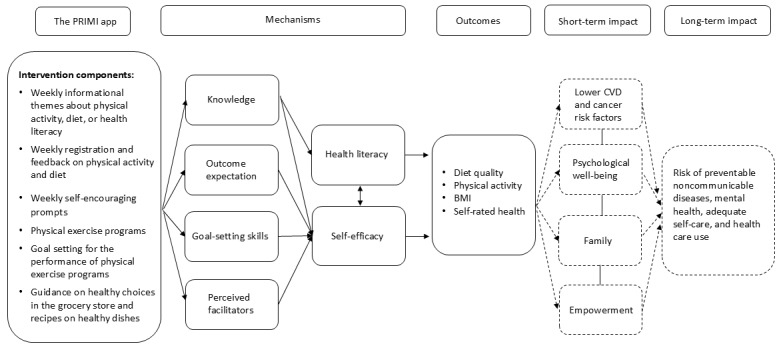
Logic model of how the components of the PRIMI (Promoting Reproductive Health in Migrant Women) app are assumed to activate theoretically driven mechanisms that may explain expected short- and long-term outcomes. CVD: cardiovascular disease.

Evidence-based recommendations from previous research [15,26-28] and key constructs from social cognitive theory [29] informed the app’s logic model (Figure 2), meaning that self-efficacy, outcome expectations, goals, and perceived facilitators were addressed through behavior change techniques embedded in the PRIMI app.

#### Content and Features

Table 1 provides an overview of the theoretical foundation and core features of the PRIMI app. As in our previous work [25], the app was built around themes that change each week during the 6-month intervention period. A total of 10 themes focus on diet and physical activity, and 5 themes address health literacy. All themes targeting diet and physical activity contain advice based on current guidelines [30] to address subjects such as food and health, energy and nutritional needs during breastfeeding, effects of physical activity, and how the body changes after childbirth. The health literacy themes covered topics such as how health care functions in Sweden, how to find trustworthy sources of health information, and patients’ rights in Swedish health care. The selection of topics was based on evidence suggesting that improved general health literacy can empower individuals to make informed health-related decisions [31], aligning with the issues discussed by women and professionals in the qualitative interviews. The vast majority of the themes include pictures or videos to enhance the information.

**Table 1:** Overview of the theoretical foundation of the PRIMI (Promoting Reproductive Health in Migrant Women) app.

Theoretical construct	PRIMI content^a^
Knowledge	Information is provided through weekly themes and a library
Self-efficacy	A feature for weekly registrations and feedback on behaviors (diet and physical activity), including a progress overview, and weekly cues for self-encouragement
Outcome expectations	Prompts are embedded to encourage reflection on the potential consequences of behavior change
Goals	Instructions on how to set specific, measurable, achievable, relevant, and time-bound goals; a goal-setting feature for PRIMI exercises; and weekly visual feedback on the achievement of goals
Facilitators	Information on how to establish new habits (eg, microhabits), weekly push notifications, recipes for healthy dishes, and (video) guidance on workout programs and healthy choices at the grocery store

^a^The behavior change techniques applied were based on the Behavior Change Technique Taxonomy version 1 [32]: information about health, social, environmental, and emotional consequences of behaviors; feedback and monitoring; identification of the self as a role model; verbal persuasion about capability; focus on past success; pros and cons; goal setting (behavior); action planning; discrepancy between current behavior and goal; behavior substitution; habit formation; prompts and cues; and instructions on how to perform the behavior.

The app also has features such as goal setting, registration with feedback for diet and physical activity, prompts for positive self-talk (eg, encourage users to recognize their efforts), healthy food recipes, supermarket tours, and video-recorded workout programs. Goals refer to the number of workout programs that users want to complete in a week (ie, 1-5), and they can be easily changed by users. The registration with feedback feature includes a prompt to complete a 6-item weekly questionnaire that addresses dietary and physical activity behaviors. When completed, users access feedback that summarizes their responses in terms of diet score and total activity minutes, graphically illustrated with bars and text using a “traffic light” approach (green=reached the recommendation, yellow=almost reached the recommendation, and red=far from reaching the recommendation) as provided by the National Board of Health and Welfare in Sweden [30]. Thus, a green light for physical activity indicates that users have reported at least 150 minutes of moderate-to-vigorous physical activity for that week, whereas a green light for the diet score represents at least 75% of the maximum score based on users’ weekly consumption of fruit and vegetables, fish and seafood, and sweets and sugary drinks.

The PRIMI app additionally contains healthy recipes developed by the Swedish Food Agency (Keyhole-labeled recipes) [33], as well as international healthy dishes developed by the registered dietitians in the PRIMI group. In addition, videos aiming to facilitate health awareness during grocery shopping are provided on the app. The physical activity module contains video-recorded workout programs at 3 levels (basic, intermediate, and advanced) with a Tabata-style interval format.

All content is free and available for all users, who can choose to engage with the app as preferred. Intended use will be defined as at least one engagement session per week where users complete the weekly registration, receive feedback on their health behaviors, and access the weekly theme. Users who accept push notifications will receive reminders 2 to 3 times per week depending on their preferences.

### Study Design

**Figure 3:**
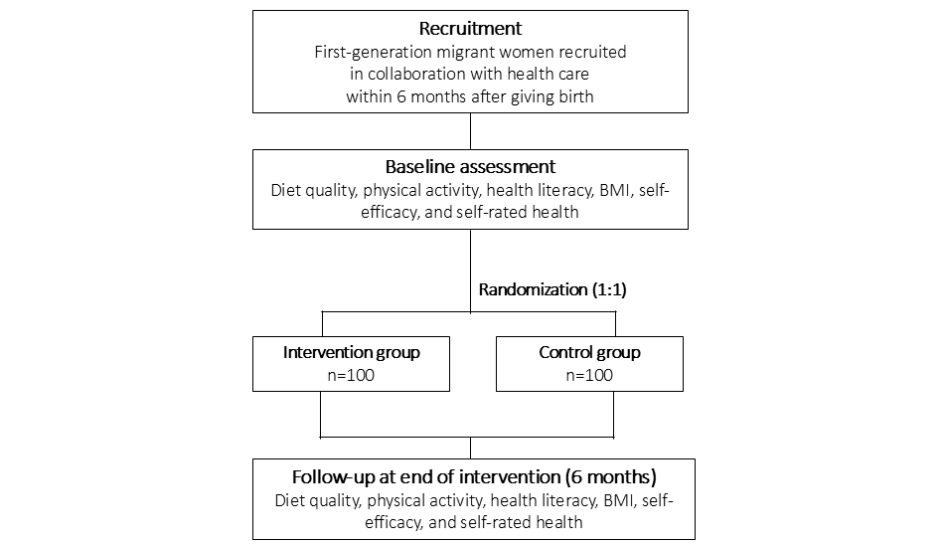
Study design of the PRIMI (Promoting Reproductive Health in Migrant Women) trial.

The PRIMI trial is a 6-month 2-arm randomized controlled superiority trial (1:1 ratio) examining the effectiveness of an mHealth intervention (the PRIMI app) after childbirth on diet quality, and moderate-to-vigorous physical activity (primary outcome) as well as health literacy, BMI, self-efficacy, and self-rated health (secondary outcome) among first-generation migrant women compared with a waitlist control condition (Figure 3). Stakeholders, including migrant women and health professionals, were engaged during the development of the PRIMI app.

**Figure 4:**
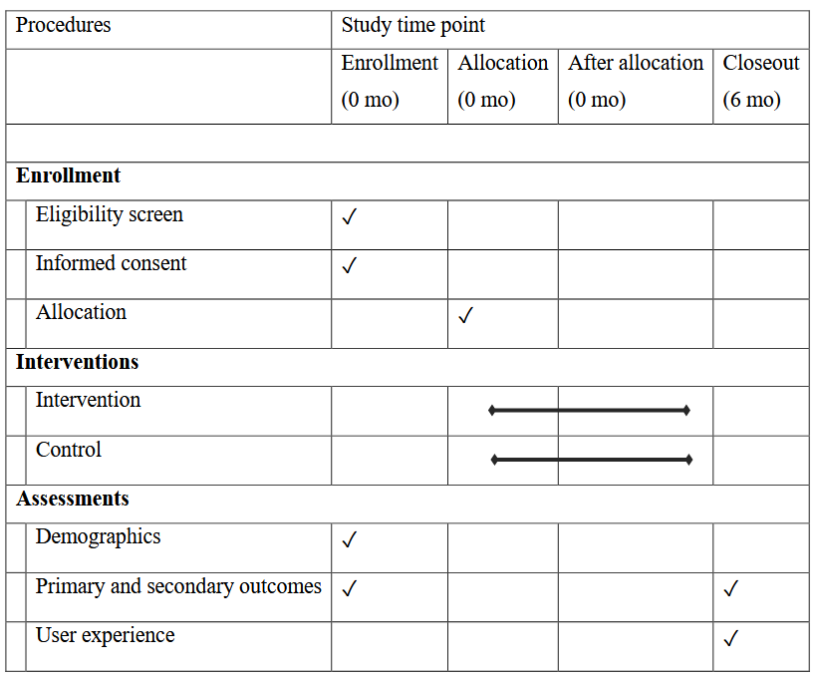
SPIRIT (Standard Protocol Items: Recommendations for Interventional Trials) table of enrollment, intervention, and assessment timeline.

The intervention group will receive standard care plus the PRIMI app for 6 months, whereas the participants in the control group will receive standard care but will be offered the PRIMI app after the 6-month follow-up (waitlist control strategy). Standard antenatal care in Sweden is free of charge for both Swedish-born and migrant women. It typically includes 8 to 9 routine visits during pregnancy and 1 to 2 postpartum visits. After childbirth, care is transferred to child health services, which monitor the development of the child and provide support to the family. This study protocol follows the SPIRIT (Standard Protocol Items: Recommendations for Interventional Trials) 2025 statement [34] (Multimedia Appendix 1), and the PRIMI trial will be reported according to the CONSORT-EHEALTH (Consolidated Standards of Reporting Trials of Electronic and Mobile Health Applications and Online Telehealth) checklist [35]. Figure 4 presents the outline of the PRIMI trial. Study outcomes will be measured at baseline and at the follow-up 6 months after randomization.

### Eligibility and Recruitment

Eligible women will be recruited through health care (eg, maternal and child health care) throughout Sweden. Inclusion criteria are first-generation migrant women who have given birth ≤6 months before, aged ≥18 years, and who would prefer to receive health-related information in any of the provided languages (ie, Arabic, Somali, English, and Swedish). No exclusion criteria will be applied.

Health care professionals will briefly inform eligible women about the trial. Women who express interest will receive additional information, including contact details for the research team. Informed consent will be obtained by health care professionals during subsequent visits or via the women returning signed consent forms. Inclusion criteria will be formally assessed based on information provided in the baseline questionnaire, which will be delivered digitally to participants via their mobile phones after they have given informed consent.

Recruitment will begin in September 2025, at 3 sites across the regions of Sweden, with each site involving 5 to 10 units. Health professionals (eg, nurses) at each unit will be introduced to the study and its procedures through in-person visits by research team members, who will provide ongoing support throughout the recruitment period. We will continue recruitment until the required number of participants has been reached. However, if recruitment is slow, we will consider additional sites and avenues other than health care channels, such as nongovernmental organizations, associations, and social media.

### Randomization and Blinding

Women will be randomly allocated to the intervention or control group after completion of the baseline measurements in a 1:1 ratio (with random block sizes of 2 and 4 to avoid revealing the allocation sequence). The *blockrand* package in the R software (version 4.5; R Foundation for Statistical Computing) will be used for the computerized random number generator. Randomization will be conducted by a statistician not part of the research team, who will remain separate from the research team members responsible for informing participants of their allocation via phone to conceal the allocation sequence. Participants and outcome assessors will not be blinded to the allocation, but the project statistician and analysts will be blinded to group allocation until completion of the analysis. Although the participants and outcome assessors are not blinded to group allocation, steps have been taken to reduce the risk of performance and detection bias. Thus, the self-reported outcomes will be collected using standardized electronic questionnaires in the preferred languages of the study participants, reducing assessor influence. When necessary, telephone-based data collection will follow a standardized script to ensure consistency across measurements.

### Control Group

Participants allocated to the waitlist control condition will receive standard care. For example, this includes regular visits to a nurse within child health care focusing on child development, vaccinations, parental guidance, and support [36]. Control group participants will be provided access to the PRIMI app upon completing the 6-month follow-up. The choice of comparator was based on the fact that no apps targeting health behaviors after childbirth are currently implemented in routine care in Sweden, as well as the lack of a suitable counterpart (ie, a similar evidence-based app) that could serve as an active control condition [37].

### Intervention Group

Women assigned to the intervention group will receive the PRIMI app in addition to standard care. After randomization, intervention group participants will be contacted via phone by a research team member who speaks Arabic, Somali, English, or Swedish based on the participant’s preference to guide them through the installation of the PRIMI app.

### Primary Outcomes

#### Diet Quality

Dietary intake will be assessed using a modified version of the National Board of Health and Welfare’s survey of health behaviors that has been previously translated and used for Arabic-, Somali-, and English-speaking women and men [25]. We will create a composite dietary quality score using four key dietary indicators based on self-reported data collected through questions addressing intake of (1) vegetables, (2) fruits and berries, (3) sweet beverages, and (4) sweet and savory snacks (Multimedia Appendix 2). The composite score will be calculated using the normalized sum of *z* scores for the 4 dietary indicators, with the scores for intake of sweet beverages and sweet and savory snacks inverted to reflect their negative health effects. Thus, a higher score will indicate better diet quality. A dietary score was assumed to better reflect participants’ overall dietary habits rather than individual dietary components.

#### Moderate-to-Vigorous Physical Activity

Women will report physical activity using a modified version of the physical activity questions in the National Board of Health and Welfare’s survey (Multimedia Appendix 2) of health behaviors, which we have previously translated into Arabic, Somali, and English [25]. The survey asks about time spent in moderate and vigorous physical activity separately, and we will combine these data to derive moderate-to-vigorous physical activity as national recommendations on physical activity consider moderate and intense activity combined.

### Secondary Outcomes

#### Health Literacy

Participants will report on their health literacy using the 16-item version of the Health Literacy Survey Questionnaire [38,39], which examines the ability to access, understand, appraise, and apply health information. Participants’ electronic health literacy (ie, ability to access, understand, appraise, and apply electronic health information) will be measured using the eHealth Literacy Scale [40].

#### BMI Measurement

Women will report their weight and height, and BMI will be calculated as weight (kg) divided by height squared (m^2^) and will be used as a continuous measure in the analyses. We will also classify BMI as underweight (<18.5 kg/m^2^), normal weight (18.5-24.9 kg/m^2^), overweight (25.0-29.9 kg/m^2^), and obesity (≥30.0 kg/m^2^).

#### Self-Efficacy

A single-item question will be used to measure self-efficacy for following a healthy diet and physical activity. Using an 11-point Likert scale from 0 (“not at all”) to 10 (“very much”), participants will be asked how confident they are in following a healthy diet and being regularly physically active given that they want to do that.

#### Self-Rated Health

Self-rated health will be assessed using the EQ-5D visual analogue scale [41], where participants rate their overall health on a scale from 0 (“worst imaginable health”) to 100 (“best imaginable health”).

#### Additional Individual Components of Diet and Physical Activity Outcomes

In addition to the composite diet quality score (primary outcome), we will examine the effectiveness of the PRIMI app on the 4 key diet indicators individually as secondary outcomes (ie, intake of vegetables, fruits and berries, sweet beverages, and sweet and savory snacks). Furthermore, beyond analyzing the effects on moderate-to-vigorous physical activity (primary outcome), we will also assess vigorous and moderate physical activity separately as secondary outcomes. All behavioral measures are presented in Multimedia Appendix 2.

#### Process Evaluation

User experience data will be collected at the 6-month follow-up by asking intervention group participants about their experiences of the PRIMI app. We will also conduct a process evaluation based on qualitative data collected through interviews with a subsample of women to obtain richer data and a deeper understanding of how the mHealth intervention was perceived and appreciated by the women [42-45].

Furthermore, automatically collected use data will be used to investigate how the app was used by participants, including the number of sessions, the number of registrations, and the time spent on the app.

#### Demographic Variables

Women will report demographic and descriptive variables such as age, educational attainment, birth country, parity, time since childbirth, and adverse pregnancy outcomes during their last pregnancy.

### Statistical Analysis and Power Considerations

Intervention effects will be analyzed according to the intention-to-treat principle using mixed linear regression models (with a random level for the individual) examining the effect of the group allocation (intervention vs control) on the primary and secondary outcomes. Data analyses will be conducted with available data as well as with missing data imputed using multiple imputations [46]. The primary analysis is the intervention effect on diet quality and moderate-to-vigorous physical activity at the 6-month follow-up adjusted for baseline values as we have done previously [20,21,25] as this procedure has the advantage of being robust to imbalances at baseline and regression toward the mean [47]. Furthermore, all models will be adjusted for age and time since childbirth. We will also consider adjusting for educational attainment and birth region provided that the sample size and distribution of these variables allow for meaningful adjustment (ie, empirical positivity).

We will also conduct a secondary per-protocol analysis including women who have used the PRIMI app (ie, engaged in at least one session) during at least 5 of the 26 intervention weeks. We will also examine whether certain variables moderate (eg, age, parity, educational attainment, birth region, self-efficacy, and health literacy) or mediate (eg, self-efficacy and health literacy) the effect of the PRIMI app on the outcomes.

A total of 100 women (50 per group) will provide at least 80% power (α=.05) to detect a minimally clinically relevant effect size of a 0.35-SD difference in the primary and secondary outcomes; we simulated trials with different sample sizes and parameter variance (*k*=100 simulations per specification) to find a suitable level whereby there was at least 80% power to detect the relevant effect [48]. It may be argued that smaller effect sizes than this may have limited clinical value, and thus, we consider the sample size sufficient. We aim to recruit 200 women (100 per group) to account for a potential dropout rate of up to 50%. However, we may consider recruiting fewer participants if the actual dropout rate is lower. To prevent dropout, participants will receive a mobile phone SMS text message reminding them about the 6-month follow-up questionnaire a few days before the questionnaire link is sent. Participants who have not completed the questionnaire within a few days will be contacted via phone to be assisted in completing it.

### Ethical Considerations

We are not aware of any potential harms related to the PRIMI app. The intervention is designed to promote a healthy diet and physical activity in line with current guidelines and is based on our previous work [20,21]. Furthermore, as we hypothesize that the PRIMI app will have health benefits, it may pose an ethical dilemma not to provide the app to the control group as well. To address this, we will apply a waitlist control group strategy, and women in the control group will be offered the intervention after the trial has ended. This is considered an ethically reasonable choice of comparator. In summary, we believe that the PRIMI project is ethically motivated as there are potential health benefits and no known harms involved. Nevertheless, it is possible that the app may be perceived as stressful among some participants. Such information will be gathered through the process evaluation of user experience data.

Before giving informed consent, all participants will receive both oral and written information about the purpose of the study, its procedures, the voluntary nature of participation, and their right to withdraw from the study at any time without consequences or the need to provide a reason. All data will be anonymized before analysis and securely stored on encrypted servers for at least 10 years in accordance with Swedish legislation, accessible only to research team members involved in the analysis to protect privacy and confidentiality. No compensation will be provided for participation in the study. The PRIMI trial has received ethics approval from the Swedish Ethical Review Authority (reference 2022-06733-01 and 2024-00135-02) and is being conducted in accordance with the Declaration of Helsinki. Any deviations from what is described in this study protocol will be reported on ClinicalTrials.gov and in the manuscript reporting the results of the trial.

## Results

Enrollment of study participants is planned to begin in September 2025. We anticipate that the study recruitment will be finalized in 2026 and that the results of the PRIMI trial will be available in 2027. Trial results will be communicated to participating health care units through in-person presentations and written reports. The results will be additionally disseminated through scientific publications, as well as through academic and clinical national and international conferences.

## Discussion

### Expected Findings

The PRIMI trial will examine whether a novel smartphone app (the PRIMI app) can improve diet quality, moderate-to-vigorous physical activity, health literacy, BMI, self-efficacy, and self-rated health in migrant women after childbirth. The PRIMI trial has several strengths, including the randomized controlled trial design and the broad inclusion criteria, which will increase the generalizability of the findings. Furthermore, the development of the PRIMI app incorporated behavior change theory [29] and empirical evidence [15,26,27] and was informed by the target population as well as practical experience from implementers (ie, health care professionals) [49] to foster acceptability and enhance the potential for positive outcomes. In addition, the various types of data collected on participants’ use and experience of the PRIMI app allow for comprehensive investigations into how and why the app may or may not be effective.

However, the conceptualization of migrant women is not straightforward as there is great heterogeneity among groups of people who may share a language but not a culture. Therefore, using language as a proxy for culture may limit the ability to address issues such as values shared within a culture. This is a limitation as the main purpose of targeting is to make messages more effective by incorporating inclusive elements [18]. Nevertheless, focusing on first-generation migrant women without applying major exclusion criteria makes the app available to more women and increases generalizability.

The PRIMI trial has several other limitations that should also be considered. First, the primary outcome measures will be self-reported by the study participants. Although the questions are based on established screening questions from the National Board of Health and Welfare and we have used them in the target group previously [25], they may be subject to recall and social desirability bias. Nevertheless, the use of the categorical answer mode has demonstrated greater validity than open-ended answers [50]. Furthermore, although participants in both the intervention and control groups will self-report outcome measures, there is a risk of detection bias as participants who do not complete the follow-up questionnaire will be contacted by the research staff, potentially revealing their group allocation. While we believe that this procedure outweighs the limitations of a higher attrition rate, we will carefully register cases in which follow-up data were collected via phone. Respondent burden was additionally considered when developing the questionnaires. Finally, the intervention period and follow-up of 6 months may be insufficient to detect long-lasting effects on health behaviors, although many mHealth interventions have had shorter intervention periods [51].

The PRIMI trial will provide novel evidence on whether an mHealth app can promote a healthy lifestyle after childbirth among migrant women. This is highly relevant given the scarcity of comparable studies on similar evidence-based mHealth interventions and the critical importance of the period after childbirth for establishing long-term health behaviors. According to a scoping review on interventions designed for pregnant women or mothers of young children to address risk factors for preventable noncommunicable diseases through health literacy, few interventions used technologies for delivery [52]. In fact, none of the included interventions (N=25) that used a mobile phone app for delivery (n=2) targeted women after pregnancy; they focused exclusively on women during pregnancy [53,54]. Furthermore, this review emphasized that interventions specifically targeting vulnerable populations, including mothers from culturally and linguistically diverse backgrounds, are highly needed [52].

### Conclusions

If the PRIMI app proves effective, it holds potential for large-scale implementation as an evidence-based intervention program at the national level. This is particularly promising given the widespread access to smartphones regardless of socioeconomic background or migrant status.

## Data Availability

Data sharing is not applicable to this paper as no datasets were generated or analyzed during this study.

## Appendix

Multimedia Appendix 1 SPIRIT (Standard Protocol Items: Recommendations for Interventional Trials) checklist.

Multimedia Appendix 2 Primary outcome measures.

## Abbreviations

CONSORT-EHEALTH: Consolidated Standards of Reporting Trials of Electronic and Mobile Health Applications and Online Telehealth
mHealth: mobile health
PRIMI: Promoting Reproductive Health in Migrant Women
SPIRIT: Standard Protocol Items: Recommendations for Interventional Trials
