# Supplementary material for: A Smartphone App (PRIMI) to Promote Healthy Diet, Physical Activity, and Health Literacy After Childbirth Among Migrant Women: Protocol for a Randomized Controlled Trial
Source: JMIR Res Protoc. 2025 Oct 17;14:e79277. doi: 10.2196/79277 (PMC12579289; doi:10.2196/79277)
Supplement: Multimedia Appendix 2 [file resprot_v14i1e79277_app2.docx]

### Primary Outcome Measures

How many 100 g portions (equivalent to an average sized banana or one large apple) of fruit did you consume **last week**?

0

1-2 portions **per week**

3-4 portions **per week**

5-6 portions **per week**

1.0 portion **per day**

1.5 portions **per day**

- 1. portions **per day**

2.5 portions **per day**

3.0 portions **per day or more**

How many 100 g portions (equivalent to an average handful) of vegetables did you consume **last week**?

0

1-2 portions **per week**

3-4 portions **per week**

5-6 portion **per week**

1 portion **per day**

- 1. portions **per day**

2 portions **per day**

2.5 portions **per day**

3 portions **per day or more**

How many cans (33 cl, one standard can) of sweet beverages (e.g. soft drinks, “energy drinks”, and cordial) did you consume **last week**? If you drank from a bottle or a glass, try to estimate roughly how much that was.

0 cans

1 can **per week**

2-3 cans **per week**

4-6 cans **per week**

1 can **per day**

1.5 cans **per day**

2.0 cans **per day**

2.5 cans **per day**

3.0 cans **per day or more**

How many portions of candy, chocolate, pastry (e.g. buns, muffins, cookies), ice cream and salty snacks (e.g. crisps, nuts, chees doodles) did you eat **last week**? *One portion is 50 g candy (9 pieces), 40 g chocolate (6 pieces/squares), 1 bun, 2 dl (scoops) of ice cream or 2 dl snacks (40 g)*.

0 portions

1 portion **per week**

2-3 portions **per week**

4-6 portions **per week**

1 portion **per day**

1.5 portions **per day**

2 portions **per day**

2.5 portions **per day**

3 portions **per day**

How much time in total did you spend on vigorous physical activity (i.e. activities producing fast increases in breathing or heart rate), for instance jogging, doing aerobics, or playing ball games, **last week?** Sum up all the time from **last week**.

0

Less than 30 minutes

30-60 minutes

1 hour

1.5 hours

2 hours

2.5 hours

3 hours

3.5 hours (i.e. 30 minutes per day)

4 hours

4.5 hours

5 hours

How much time in total did you spend on moderate physical activity (e.g. walking, cycling, or gardening) **last week**? Sum up all the time from **last week** (at least 10 minutes at a time).

0

Less than 30 minutes

30-60 minutes

1 hour

1.5 hours

2 hours

2.5 hours

3 hours

3.5 hours (i.e. 30 minutes per day)

4 hours

4.5 hours

5 hours

5.5 hours

6 hours

6.5 hours

7 hours (i.e. 1 hour per day)
